# Supplementary material for: Molecular Characteristics and Genetic Evolution of Echovirus 33 in Mainland of China
Source: Pathogens. 2022 Nov 18;11(11):1379. doi: 10.3390/pathogens11111379 (PMC9697921; doi:10.3390/pathogens11111379)
Supplement: Supplementary file 1 [file pathogens-11-01379-s001.zip › pathogens-1970583 - Supplementary.pdf]

Supplementary materials

Table S1. Information of these 7 patients involved in this study.

ID	Gender	Age	Province	Collected time	Diagnosis	clinical classification
**2013HUN-019**	female	62	HuNan	2013-06-26	Influenza-like illness	/
**2013HUN-037**	male	7	HuNan	2013-06-21	HFMD	mild case
**2015JS-087**	female	1	Jiangsu	2015-03-13	HFMD	mild case
**2015JS-088**	female	2	Jiangsu	2015-04-15	HFMD	mild case
**2015JS-089**	male	3	Jiangsu	2015-03-31	HFMD	mild case
**2018XJ-113**	female	6	Xinjiang	2018-05-22	HFMD	mild case
**2018XJ-115**	female	5	Xinjiang	2018-06-17	HFMD	mild case

Table S2. E33 full-length sequencing primers.

Name	Location	Primer sequence(5′-3′)	Orientation	Soure
0001S48	-	GGGGACAAGTTTGTACAAAAAAGCAGGCTTTAAAACA GCTCTGGGGTT	Forward	[22]
EVP4	529-548	CTACTTTGGGTGTCCGTGTT	Forward	[23]
E33-871R	852-871	CTATTGGCAGAATTCGATGC	Reverse	This study
E33-1071F	1071-1090	TATCTCAAGGACGACGAGGC	Forward	This study
OL68-1	1159-1178	GGTAAYTTCCACCACCANCC	Reverse	[23]
E33-1571F	1571-1590	TGCCTTACATCAACAGCGTC	Forward	This study
E33-1890R	1871-1890	ACTACCGAGTCCACTTCTGC	Reverse	This study
E33-2435F	2435-2454	GTACATGATGCTGTTGTTGG	Forward	This study
E33-2613R	2594-2613	TGTGCACATGCCTAGTTTGC	Reverse	This study
E33-3169F	3169-3188	CCGTTTCCAGTGTAGTGAGA	Forward	This study
E33-3273R	3254-3273	CCTGTTGTCATTAAATCCGC	Reverse	This study
E33-3950F	3950-3969	GACCTAATTACAGTGACCGC	Forward	This study
E33-4095R	4076-4095	GCGTTGGTCATCTCTGTGAA	Reverse	This study
E33-4656F	4656-4675	TAGTGTTGACTTTGTACCGC	Forward	This study
E33-4924R	4905-4924	CTGGATGGCTTTGCCACATA	Reverse	This study
E33-5466F	5466-5485	AGA GTA CGG TGA ATT CAC GA	Forward	This study
E33-5608R	5589-5608	TTGAGTTTCAGGAGTGTCAG	Reverse	This study
E33-6209F	6209-6228	ACAGCTAGCAACCTTAGACA	Forward	This study
E33-6427R	6408-6427	GATCGTAGTTCATCCTTCAC	Reverse	This study
E33-6609F	6609-6628	TGATTACAGTGGGTATGACG	Forward	This study
E33-6882R	6853-6882	CACCTTCAGCATTAGTGTTC	Reverse	This study
7500 A	-	GGGGACCACTTTGTACAAGAAAGCTGGG(T)24	Reverse	[22]

Table S3. 106 isolates used for phylogenetic analysis of E33.

No	genotype	GenBank Accession	Sample ID	Isolation Country	Isolation Year	Source
1	A	AJ241436.1	Toluca-3	France	1959	GeneBank
2	B	AY167758.1	NEZ84-1967	New-Zealand	1984	GenBank
3	B	AY167759.1	NEZ84-2723	New-Zealand	1984	GenBank
4	B	AY167760.1	NEZ83-7709	New-Zealand	1983	GenBank
5	B	AY167761.1	NEZ83-7776	New-Zealand	1983	GenBank
6	B	AY167762.1	NEZ84-3401	New-Zealand	1984	GenBank
7	B	AY167763.1	NEZ84-3303	New-Zealand	1984	GenBank
8	C	AY167764.1	AK-30.04.00	New-Zealand	2000	GenBank
9	C	AY167765.1	WHANG-03.09.00	New-Zealand	2000	GenBank
10	C	AY167766.1	WK-29.04.00	New-Zealand	2000	GenBank
11	C	AY167767.1	WK-18.04.00	New-Zealand	2000	GenBank
12	C	AY167768.1	NL-13.05.00	New-Zealand	2000	GenBank
13	C	AY167769.1	WK-25.03.00	New-Zealand	2000	GenBank
14	C	AY167770.1	HU-11.08.00	New-Zealand	2000	GenBank
15	C	AY167771.1	WK-05.05.00	New-Zealand	2000	GenBank
16	C	AY167772.1	WK-04.05.00	New-Zealand	2000	GenBank
17	C	AY167773.1	AK-19.05.00	New-Zealand	2000	GenBank
18	C	AY167774.1	WK-18.04.00W	New-Zealand	2000	GenBank
19	C	AY167775.1	AK-29.05.00	New-Zealand	2000	GenBank
20	C	AY167776.1	WK-14.05.00	New-Zealand	2000	GenBank
21	C	AY167777.1	HU-13.07.00	New-Zealand	2000	GenBank
22	C	AY167778.1	AK-03.05.00	New-Zealand	2000	GenBank
23	C	AY167779.1	WK-11.05.00	New-Zealand	2000	GenBank
24	C	AY167780.1	WK-11.04.00	New-Zealand	2000	GenBank
25	C	AY167781.1	WK-26.04.00	New-Zealand	2000	GenBank
26	C	AY167782.1	AK-01.06.00	New-Zealand	2000	GenBank
27	C	AY167783.1	HU-24.08.00	New-Zealand	2000	GenBank
28	C	AY167784.1	WN-06.11.00	New-Zealand	2000	GenBank
29	C	AY167785.1	WN-13.06.00	New-Zealand	2000	GenBank
30	C	AY167786.1	WN-20.09.00	New-Zealand	2000	GenBank
31	C	AY167787.1	WK-05.05.00W	New-Zealand	2000	GenBank
32	C	AY167788.1	WK-30.04.00	New-Zealand	2000	GenBank
33	C	AY167789.1	WK-06.10.00	New-Zealand	2000	GenBank
34	C	AY167790.1	WK-28.08.00	New-Zealand	2000	GenBank
35	C	AY167791.1	WK-29.04.00J	New-Zealand	2000	GenBank
36	C	AY167792.1	WK-28.04.00	New-Zealand	2000	GenBank
37	C	AY167793.1	WK-21.07.00	New-Zealand	2000	GenBank
38	C	AY167794.1	WN-05.09.00	New-Zealand	2000	GenBank
39	C	AY167795.1	AK-04.05.00	New-Zealand	2000	GenBank
40	C	AY167796.1	NL-02.07.00	New-Zealand	2000	GenBank
41	E	KT965725.1	YNK35/CHN/2013	China	2013	GenBank
42	E	KU379646.1	YNA12/CHN/2013	China	2013	GenBank
43	C	ON873778	13019/HuN/CHN/2013	China	2013	This study
44	C	ON873779	13037/HuN/CHN/2013	China	2013	This study
45	C	ON873780	15087/JS/CHN/2015	China	2013	This study
46	C	ON873781	15088/JS/CHN/2015	China	2013	This study
47	C	ON87382	15089/JS/CHN/2015	China	2013	This study
48	C	ON873783	18113/XJ/CHN/2018	China	2018	This study
49	C	ON873784	18115/XJ/CHN/2018	China	2018	This study
50	C	AM711014.1	234024-05	France	2005	GenBank
51	C	AY167797.1	OMA98-0168	Oman	1998	GenBank
52	C	AY167798.1	OMA98-0172	Oman	1998	GenBank
53	C	AY167799.1	AUS96-2688	Australia	1996	GenBank
54	C	HF948113.1	CF235069_FRA05	France	2005	GenBank
55	D	JN203971.1	N-21	India	2008	GenBank
56	D	JN203972.1	N-22	India	2008	GenBank
57	C	JN203973.1	N-105	India	2008	GenBank
58	C	JN203974.1	N-106	India	2008	GenBank
59	C	JN203975.1	N-138	India	2008	GenBank
60	D	JN203977.1	N-152A	India	2008	GenBank

61	C	JN203978.1	N-329	India	2008	GenBank
62	C	JN203979.1	N-346	India	2008	GenBank
63	C	JN203981.1	N-420	India	2008	GenBank
64	D	JN203982.1	N-519	India	2008	GenBank
65	D	JN203983.1	N-730	India	2008	GenBank
66	C	JN203984.1	N-873	India	2008	GenBank
67	C	JN203985.1	N-977	India	2008	GenBank
68	C	JN203986.1	N-998	India	2008	GenBank
69	C	JN203987.1	N-1029A	India	2008	GenBank
70	C	JN203989.1	N-1058	India	2008	GenBank
71	C	JN203991.1	N-1069	India	2008	GenBank
72	C	JN203992.1	N-1071	India	2008	GenBank
73	C	JX513554.1	A015D	India	2008	GenBank
74	C	JX513555.1	A016D	India	2008	GenBank
75	C	JX513556.1	A022D	India	2008	GenBank
76	C	JX513557.1	A032D	India	2008	GenBank
77	C	JX513558.1	A033D	India	2008	GenBank
78	C	JX513559.1	A034D	India	2008	GenBank
79	C	JX513560.1	KCD77-1	India	2010	GenBank
80	C	JX513561.1	A93-22D	India	2010	GenBank
81	C	JX513562.1	A107-18D	India	2010	GenBank
82	C	KF177104.1	A78-34PI	India	2010	GenBank
83	C	KF177105.1	A85-28PI	India	2010	GenBank
84	C	KF177106.1	A91-20a	India	2010	GenBank
85	C	KF177107.1	A94-19a	India	2010	GenBank
86	C	KF177108.1	A103-18PI	India	2010	GenBank
87	C	KF177109.1	A103-19PI	India	2010	GenBank
88	C	KF177110.1	A108-18PD	India	2010	GenBank
89	C	KF512502.1	ILIHuN13-1	China	2013	GenBank
90	C	KF512503.1	ILIHuN13-2	China	2013	GenBank
91	C	KF512504.1	ILIHuN13-3	China	2013	GenBank
92	C	KF512505.1	ILIHuN13-4	China	2013	GenBank
93	C	KF512506.1	ILIHuN13-5	China	2013	GenBank
94	C	KF512507.1	ILIHuN13-6	China	2013	GenBank
95	C	KF512508.1	ILIHuN13-7	China	2013	GenBank
96	C	KF512509.1	ILIHuN13-8	China	2013	GenBank
97	C	KF512510.1	ILIHuN13-9	China	2013	GenBank
98	C	KP638484.1	HB92	China	2014	GenBank
99	C	KU574620.1	PMKA1174/THA/2011	Thailand	2011	GenBank
100	C	KU574640.1	PMKA0914/THA/2010	Thailand	2010	GenBank
101	C	LC120901.1	61-YN-CHN-2014JK	China	2014	GenBank
102	C	LC120903.1	62-YN-CHN-2014JK	China	2014	GenBank
103	C	LC120904.1	64-YN-CHN-2014JK	China	2014	GenBank
104	D	LC120942.1	65-YN-CHN-2014JK	China	2015	GenBank
105	C	LT883146.1	316-YN-CHN-2015JK	China	2017	GenBank
106	D	MN018205.1	P136/ZS/CHN/2010	China	2010	GenBank

Table S4. Comparison of nucleotide sequence consistency between the representative strains of E and C genotypes and the prototype strains(AY302556.1_Toluca-3) and other Enterovirus B group.

Region	KU379646		KT965725		2013HUN-19		2015JS-87		2018XJ-113	
	E33 Prototype	Other EV-B Prototype	E33 Prototype	Other EV-B Prototype	E33 Prototype	Other EV-B Prototype	E33 Prototype	Other EV-B Prototype	E33 Prototype	Other EV-B Prototype
5'-UTR	82	65.4-87	81.5	65.1-86.5	82.4	66.4-88.8	81.8	65.6-87.9	82.1	66.1-88.5
VP4	79.2	68.5-82.1	79.2	68.5-82.1	80.6	68.5-84	80.6	67.1-82.6	78.7	67.6-84
VP2	78.9	64.5-72.7	77.5	64.4-71.5	78.5	64.9-71.2	77.7	65.1-71.7	78.2	64.9-72
VP3	78.4	62.7-72.8	78.4	62.3-72.6	79.4	63-71.8	79.6	63-72.2	78.7	61.9-70.8
VP1	78.1	52.8-68.4	78.1	52.8-68.4	76.3	54.2-66.7	76.2	54-66.5	76.2	54.7-67.4
2A	76.8	73.5-84.4	76.8	73.5-84.4	78	73.3-82.4	77.5	74-81.1	78.4	73.3-81.3
2B	76	75-84.1	76	75-84.1	76.7	74.7-85.1	77.1	74.7-84.8	76.7	74.7-85.1
2C	80.8	78.9-84.3	80.9	79-84.4	80.8	79.6-85.1	81	79.1-85.6	80.3	78.8-85.3
3A	77.9	71.9-79.7	81.2	74.5-82.7	79	74.9-84.2	78.6	74.9-84.2	77.9	74.9-84.6
3B	68.1	66.6.-80.3	77.2	74.2-89.3	74.2	71.2-90.9	74.2	71.2-89.3	74.2	72.7-87.8
3C	79.2	74.6-81.9	80.8	76.6-84.1	77.5	75.5-84.6	76.3	75.4-85.2	77.4	74.6-84.8
3D	81.3	78.1-83.9	81.3	78.2-84	79.2	78-86	78.5	77-85.8	78.7	77.7-86
3'-UTR	79.8	73.7-86.5	82.6	70.8-88.4	85.5	78.6-92.3	84.6	77.6-91.3	86.5	79.6-92.3

P1 region

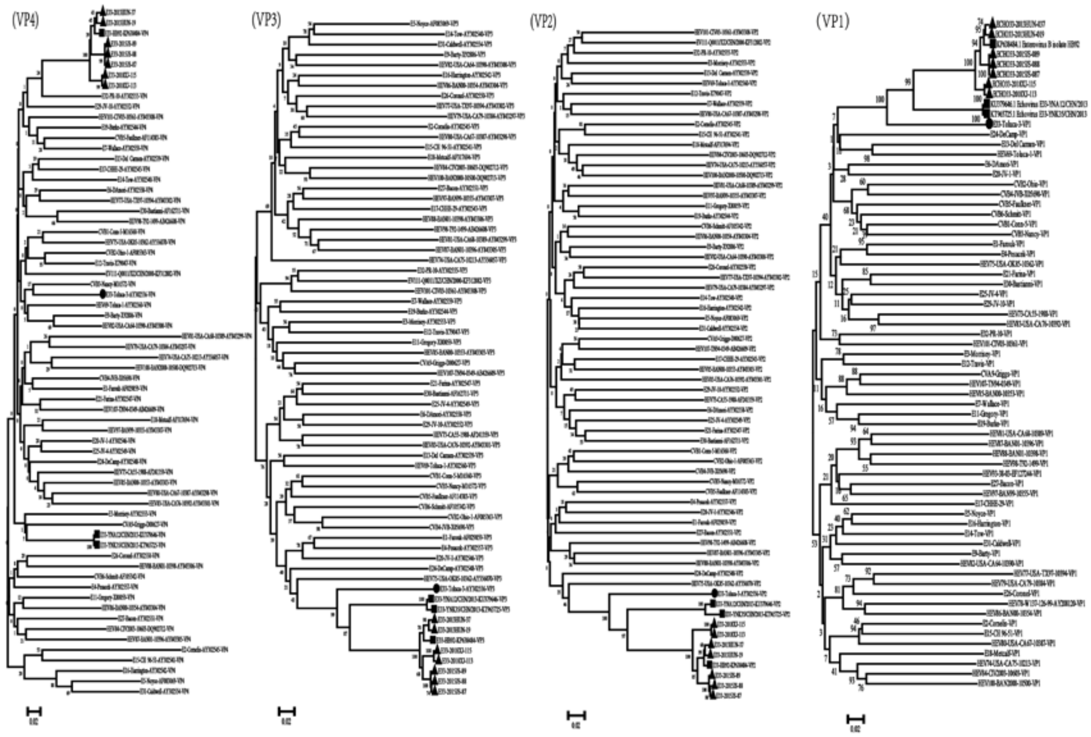

Figure S1. NJ trees based on *P1* regions of the prototype sequence of all *Enterovirus B* in the GenBank database and seven E33 strains in this study. ● indicates E33 prototype strain (Toluca-3); ▲indicates 7 strains of E33 in this study ; ■ indicates represents other E33 strains in China. Numbers on codes indicate the bootstrap support of the node (1000 bootstrap replicate percentage). Scale bars represent the genetic distance, and all panels have the same scale. (VP4) VP4 region sequences; (VP3) VP3 region sequences; (VP2) VP2 region sequences; （VP1）VP1 region sequences.

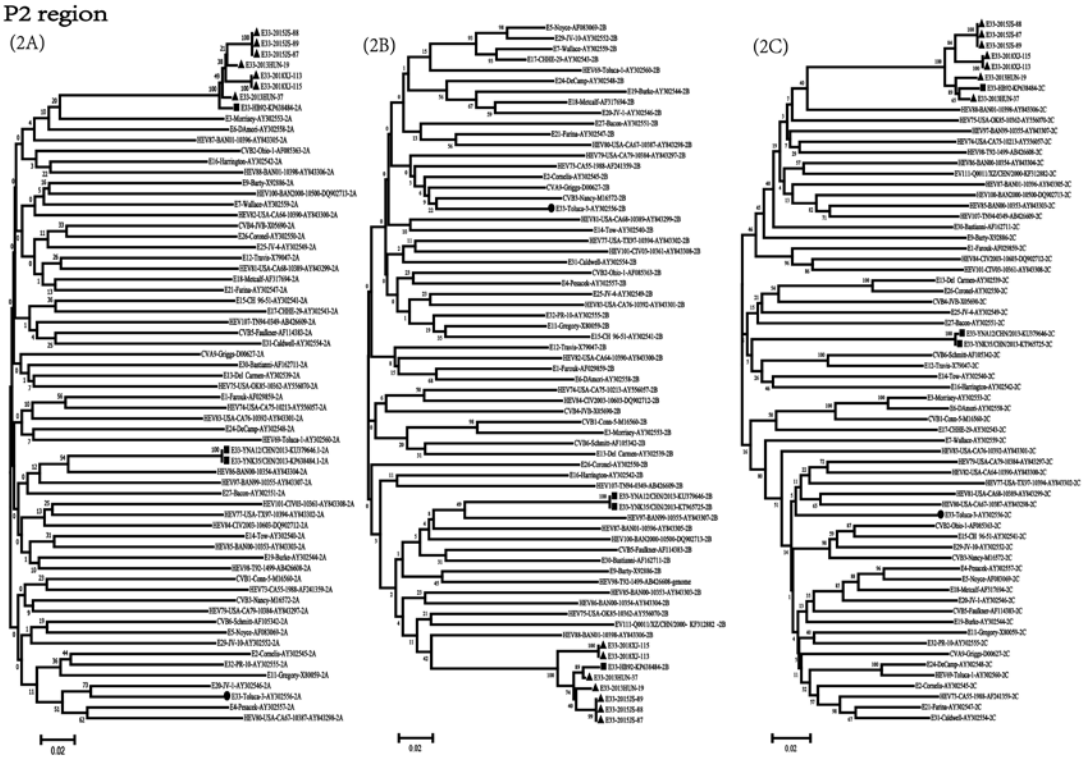

Figure S2. NJ trees based on P2 regions of the prototype sequence of all *Enterovirus B* in the GenBank database and seven E33 strains in this study. ● indicates E33 prototype strain (Toluca-3); ▲indicates 7 strains of E33 in this study ; ■ indicates represents other E33 strains in China. Numbers on codes indicate the bootstrap support of the node (1000 bootstrap replicate percentage). Scale bars represent the genetic distance, and all panels have the same scale. (2A) 2A region sequences; (2B) 2B region sequences; (2C) 2C region sequences.

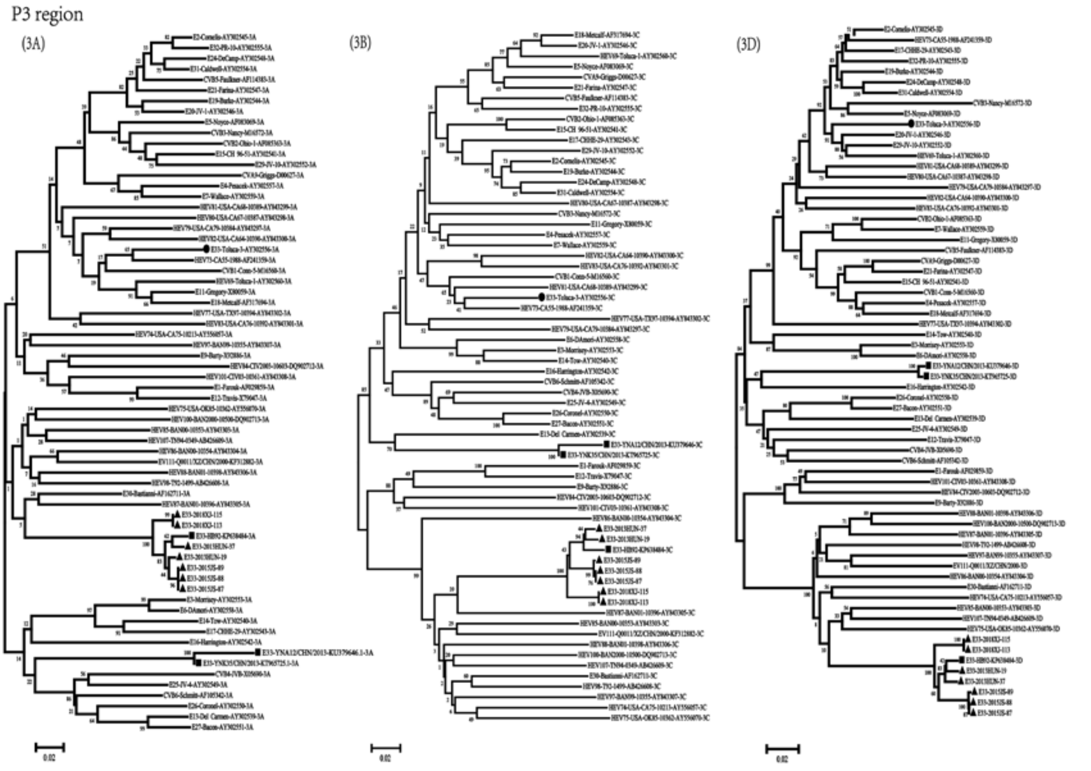

Figure S3. NJ trees based on P3 regions of the prototype sequence of all *Enterovirus B* in the GenBank database and seven E33 strains in this study. ● indicates E33 prototype strain (Toluca-3); ▲indicates 7 strains of E33 in this study ; ■ indicates represents other E33 strains in China. Numbers on codes indicate the bootstrap support of the node (1000 bootstrap replicate percentage). Scale bars represent the genetic distance, and all panels have the same scale. (3A) 3A region sequences; (3B) 3B region sequences; (3C) 3C region sequences.

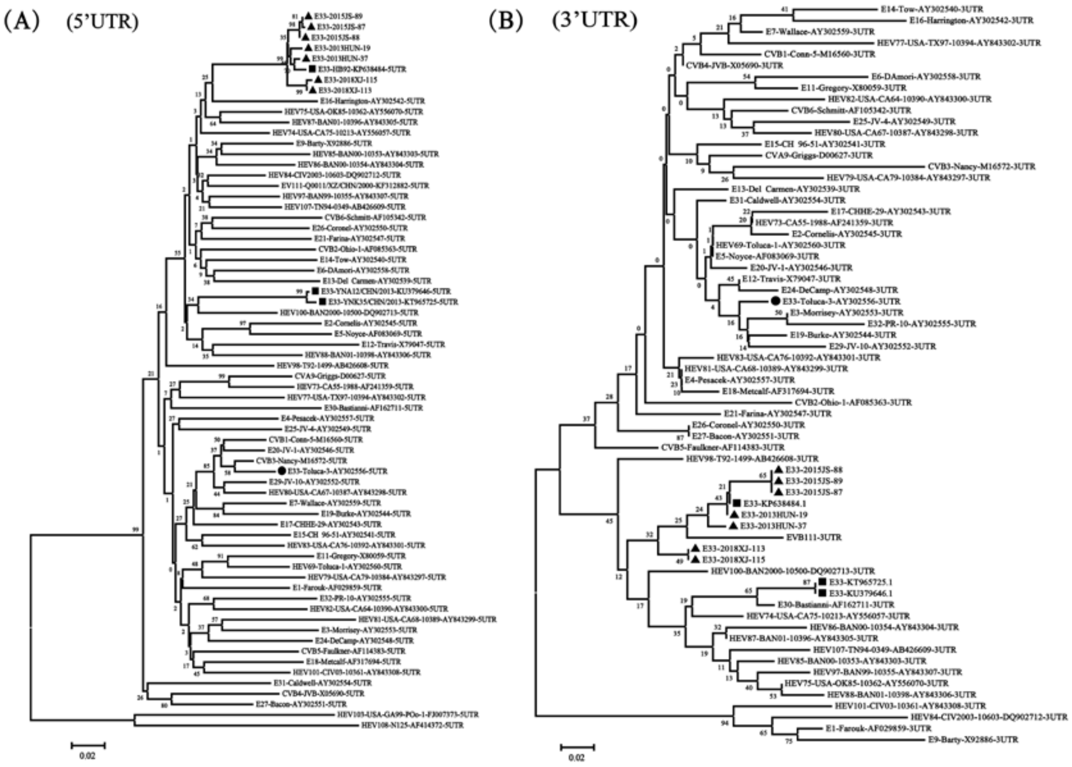

Figure S4. NJ trees based on 5′UTR, 3′UTR regions of the prototype sequence of all *Enterovirus B* in the GenBank database and seven E33 strains in this study. ● indicates E33 prototype strain (Toluca-3);▲indicates 7 strains of E33 in this study ; ■ indicates represents other E33 strains in China. Numbers on codes indicate the bootstrap support of the node (1000 bootstrap replicate percentage). Scale bars represent the genetic distance, and all panels have the same scale. (A) 5′UTR region sequences; (B) 3′UTR region sequences.

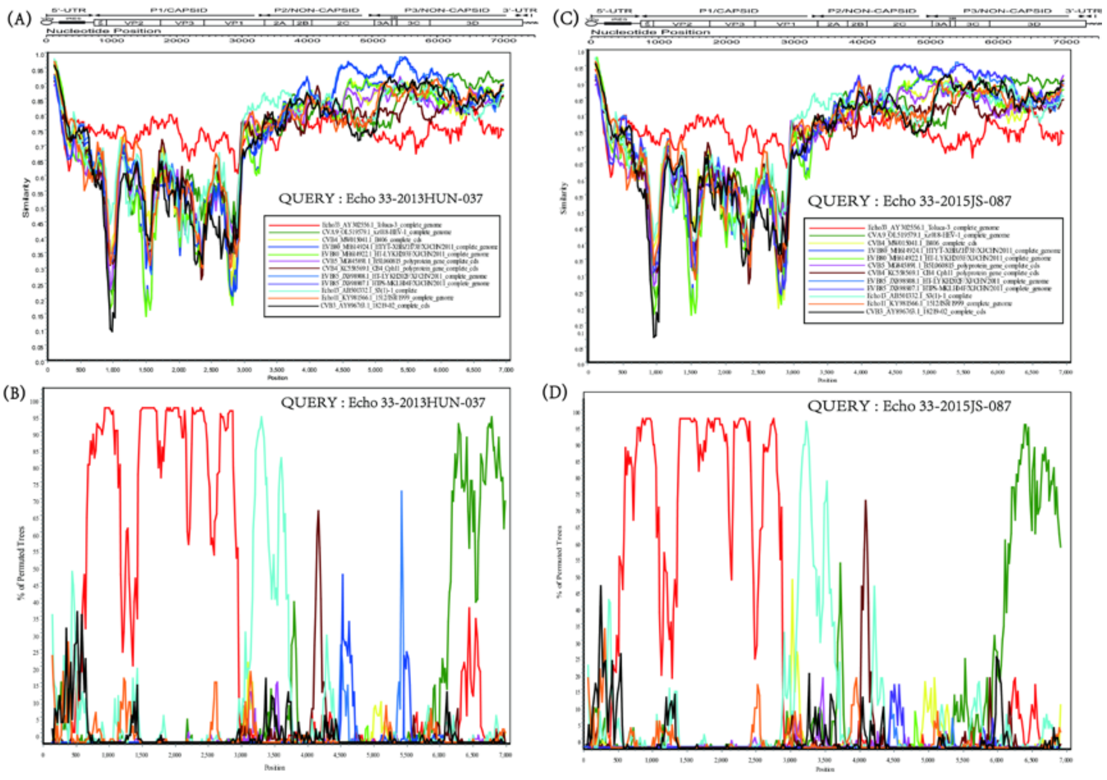

Figure S5. Recombination analyses of other two strains in this study. (A) , (B) shows separately Simplot and BootScan plot of E33-2013HUN-037 using a sliding window of 200-nt moving in 20-nt steps (C) ,(D) shows separately Simplot and BootScan plot of E33-2015JS-087.

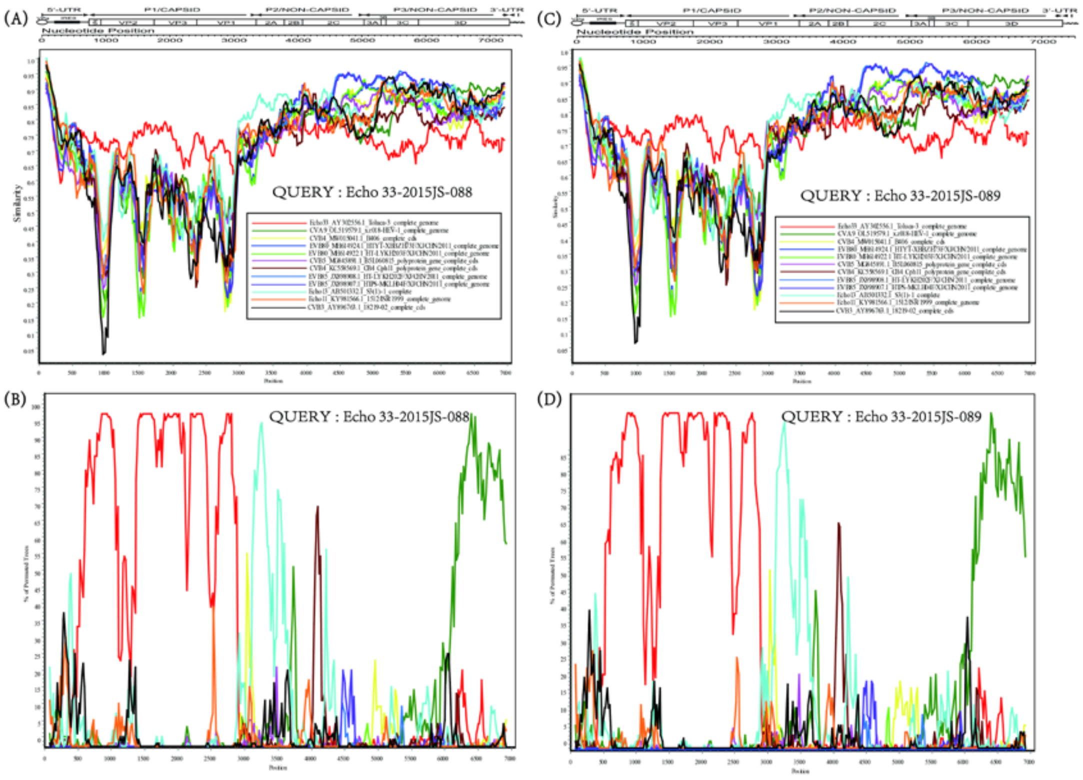

Figure S6. Recombination analyses of other two strains in this study. (A) , (B) shows separately Simplot and BootScan plot of E33-2015JS-088 using a sliding window of 200-nt moving in 20-nt steps (C) ,(D) shows separately Simplot and BootScan plot of E33-2015JS-089.

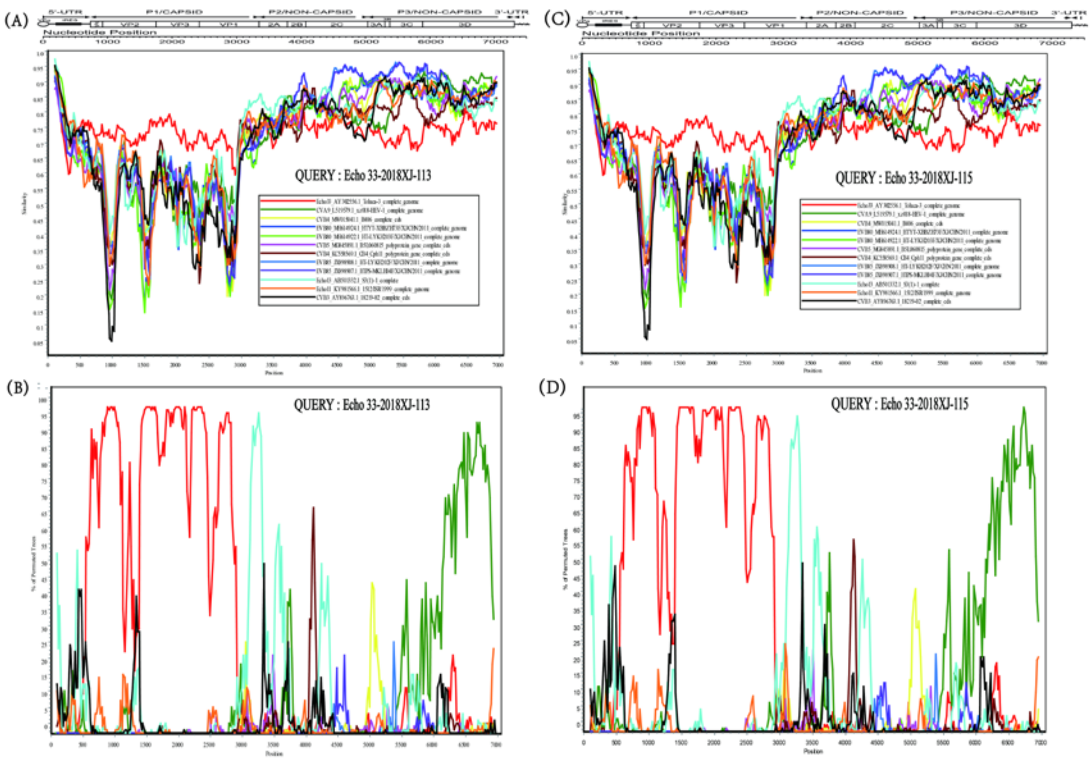

Figure S7. Recombination analyses of other two strains in this study. (A), (B) shows separately Simplot and BootScan plot of E33-2018XJ-113 using a sliding window of 200-nt moving in 20-nt steps (C), (D) shows separately Simplot and BootScan plot of E33-2018XJ-115.
